# Transcriptomic dynamics of breast cancer progression in the MMTV-PyMT mouse model

**DOI:** 10.1186/s12864-017-3563-3

**Published:** 2017-02-17

**Authors:** Ying Cai, Ruben Nogales-Cadenas, Quanwei Zhang, Jhih-Rong Lin, Wen Zhang, Kelly O’Brien, Cristina Montagna, Zhengdong D. Zhang

**Affiliations:** 1 Department of Genetics, Albert Einstein College of Medicine, Bronx, NY USA; 2 Department of Pathology, Albert Einstein College of Medicine, Bronx, NY USA

**Keywords:** RNA-sequencing, PyMT mouse model, Breast cancer

## Abstract

**Background:**

Malignant breast cancer with complex molecular mechanisms of progression and metastasis remains a leading cause of death in women. To improve diagnosis and drug development, it is critical to identify panels of genes and molecular pathways involved in tumor progression and malignant transition. Using the PyMT mouse, a genetically engineered mouse model that has been widely used to study human breast cancer, we profiled and analyzed gene expression from four distinct stages of tumor progression (hyperplasia, adenoma/MIN, early carcinoma and late carcinoma) during which malignant transition occurs.

**Results:**

We found remarkable expression similarity among the four stages, meaning genes altered in the later stages showed trace in the beginning of tumor progression. We identified a large number of differentially expressed genes in PyMT samples of all stages compared with normal mammary glands, enriched in cancer-related pathways. Using co-expression networks, we found panels of genes as signature modules with some hub genes that predict metastatic risk. Time-course analysis revealed genes with expression transition when shifting to malignant stages. These may provide additional insight into the molecular mechanisms beyond pathways.

**Conclusions:**

Thus, in this study, our various analyses with the PyMT mouse model shed new light on transcriptomic dynamics during breast cancer malignant progression.

**Electronic supplementary material:**

The online version of this article (doi:10.1186/s12864-017-3563-3) contains supplementary material, which is available to authorized users.

## Background

Breast cancer is a globally prevalent disease and a leading cause of cancer-related mortality among women of all ages [1]. In 2008 there were 1.38 million new cases worldwide, and the disease caused ~458,400 deaths [2]. The number increased to 1.67 million in 2012, accounting for 11.9% of all cancer new cases [3]. A malignancy with a multistep pathological process, breast cancer in humans starts with the premalignant atypical ductal hyperplasia (ADH), followed by ductal carcinoma *in situ* (DCIS) and subsequent malignant invasive ductal carcinoma (IDC) [4]. Patients surviving the primary tumors often die of carcinoma-culminated metastasis [1]. Despite widely recognized evidence that ADH and DCIS are precursors of IDC, few biomarkers identified from the early stages can explain and predict tumor progression. Many genes have been shown to contribute to breast cancer development [5], but the molecular mechanisms of its progression remains largely unknown, which greatly limits our abilities for early diagnosis and treatment of breast cancer patients with metastasis risk [6].

Transgenic mouse models have been widely used to study breast cancer, and the PyMT mouse model is one of them [7–9]. Expression of the oncoprotein, polyoma middle T (PyMT) antigen from mouse polyoma virus, is under the control of the mouse mammary tumor virus (MMTV) long terminal repeats (LTR) and is restricted to mammary epithelia [10]. By stimulating multiple signaling including Shc and PI3-kinase, the membrane scaffold protein PyMT activates MAPK and PI3K pathways that function in cell proliferation and survival [11, 12]. Sharing both morphological and transcriptional features with the human disease [10] and resembling the human luminal B subtype of breast cancer on gene expression profiles [9], the MMTV-PyMT transgenic mice provide us a reliable animal model for breast cancer progression. The primary tumors developed in this mouse model go through four stereotypical stages of cancer progression – hyperplasia, adenoma/mammary intraepithelial neoplasia (MIN), early and late carcinoma – while progress from pre-malignancy to malignancy.

Because most previous studies of PyMT mice focused only on the carcinoma stages, little is known about gene expression alterations in the early stages as well as their impacts to the later stages. In this study, we examined gene expression dynamics in the full range of breast tumor development from hyperplasia to late carcinoma. Using RNA sequencing instead of microarray, our data had a wider dynamic range and a higher sensitivity to better detect differentially or lowly expressed genes. Moreover, to go beyond single gene inspected by earlier studies, we explored biological networks to learn the connections and interplays among genes. Networks are powerful in interpreting the underlying mechanisms of diseases by revealing disease modules, which are groups of highly connected genes or gene products [13]. In this study, we carried out differential gene expression profiling, time-course analysis and network-based gene screening to identify candidate genes that may contribute to breast cancer progression. We found that many genes differentially expressed in the late carcinoma stage initiated the expression alteration at the hyperplasia stage. We also identified genes with disrupted expression during the transition from premalignance to malignance. Last, we found gene modules that co-expressed in tumors with hub genes predict future metastasis. Thus, by proposing novel candidate oncogenes that may promote tumor progression and malignant transition, our study helps to find genes as potential biomarkers and drug targets for breast cancer treatment.

## Methods

### Animals and tissue collection

This study of cancer in mice was approved by the Institutional Animal Care and Use Committee (IACUC) of Albert Einstein College of Medicine. All procedures involving mice were conducted in accordance with the National Institutes of Health guidelines concerning the use and care of experimental animals. Male PyMT mice (FVB/N-Tg(MMTV-PyVT)634Mul/J mice, Stock Number: 002374, the Jackson Laboratory) were randomly bred with homozygous FVB females to obtain F1 female mice (PyMT mice hereafter) heterozygous for the PyMT transgene; they developed breast cancer and were used as cases. Homozygous FVB females were used as controls.

We selected four time points corresponding to different tumor progression stages: hyperplasia at week 6, adenoma/MIN at week 8, early carcinoma at week 10, and late carcinoma at week 12 [10]. At each time point, mammary tumors and normal mammary glands were collected from three PyMT mice and three age-matched FVB controls, respectively. We snap froze all samples and kept them at –80°C. We had a pathologist examined the morphology of our PyMT tumor samples and observed typical features of various tumor developmental stages (Additional file 1: Figure S1A). We also estimated the percentage of infiltrating cells as the sample purity. By H&E staining, the pathology report showed less than 5% stromal and muscle cells, with rare inflammatory cells in carcinoma samples. In general, we had greater than 90% tumor cells in our carcinoma samples, which surpassed the Cancer Genome Atlas (TCGA) criteria (over 60% tumor cells for human tumor samples) [14, 15].

### Directional RNA sequencing

Total RNAs were extracted from frozen samples using the miRNeasy mini kit (Qiagen) according to the manufacturer’s protocol. Agilent 2100 Bioanalyzer was used to check RNA quality. The total RNA was treated with DNaseI, depleted of ribosomal RNA with Ribo-Zero Magnetic Gold Kit (Epicentre), followed by ethanol precipitation. Next, RNA was converted to cDNA using SuperScript III First-Strand Synthesis Kit (Invitrogen) with 80ng random hexamers and 50mM oligo-dT and subsequently ethanol precipitated. Single-stranded cDNA was converted to dsDNA by DNA polymerase I while incorporating dU/VTPs (10mM). Samples were fragmented to 200–300bp using Covaris. After fragmentation, samples were purified using the MinElute PCR purification kit (Qiagen). Fragmented samples underwent standard end-repair, dA-tailing and adapter ligation using Illumina TruSeq adapters for multiplexing. Adapter-ligated cDNA was treated with uracil-DNA glycosylase followed by enrichment PCR using Kapa reagents for 14 cycles. Libraries were size selected for 150–600 bp on a 2% low-melt ultra low-range agarose gel stained with SYBR Gold (Invitrogen) to eliminate adaptor dimers. Purified libraries were used to sequence on Hiseq2500 according to standard protocols. The PyMT RNA-sequencing data is available in the Gene Expression Omnibus (GEO) database as GSE76772.

### Statistical analysis

Sequence data were preprocessed by WASP 3.0 [16], an in-house pipeline. FastQC [17] was used for reads quality control. The raw FASTQ files were trimmed for adapter sequences using quart. Then GSNAP v2012-07-02 [18] was used to align reads to mm9 reference genome with default settings. Gene counts were given by HTSeq v0.5.3p3 [19].

All statistical analyses were carried out with R v3.0.1 [20]. After removing transcriptionally inactive genes (read count per million < 1 in more than half of the samples) from raw RNA-sequencing gene counts, we got high confident gene counts, which were then normalized by DESeq [21]. The PCA analysis used method adopted from DESeq. The R package edgeR v3.4.2 [22] was used to perform statistical analysis on gene counts and to detect differentially expressed genes. Differentially expressed genes (DEGs) at each stage were analyzed for enrichment on REACTOME [23] and KEGG [24] pathways using the R package GOseq [25], which corrects bias owing to gene length and expression variability. To reduce noise and redundancy, we used GSEA (Gene Set Enrichment Analysis) [26] to investigate hallmark gene sets (“Hallmark gene sets summarize and represent specific well-defined biological states or processes and display coherent expression”, as defined by GSEA).

### Weighted gene co-expression network analysis

We conducted the weighted gene co-expression network analysis (WGCNA) using the R package WGCNA [27] to identify co-expressed genes. We used the normalized expression values from aforementioned analysis as input to construct a signed gene co-expression network. Then we looked at module preservation to find the most robust and generalized modules using a composite Z-summary statistic. For highly preserved modules, we calculated the module eigengene (the first principal component). Labels that denote the disease stages (normal, hyperplasia, adenoma/MIN, early carcinoma and late carcinoma) were permuted. We permuted the labels 10,000 times, and used an R package coin to test the statistical significance of Spearman’s correlation coefficient between tumor progression stages and module eigengenes. The results were exported to VisANT [28] for network visualization.

### Time-course data analysis

To model the transitional changes in gene expression, we calculated a log2 ratio change (LRC) of each gene between stages *i* + 1 and *i* using results from the previous DEGs analysis: LRC = log_2_R^(*i*+1)^ – log_2_R^(*i*)^ (*i* = 1, 2, 3; stage 1 as hyperplasia). After normalizing LRCs to z-scores, we categorized the transitional changes into positive (+1), negative (–1), and constant (0) transition statuses by their deviance from the mean. Since there are three transitions and three transition statuses, altogether we have 3^3^ = 27 possible groups of transition patterns.

To find gene expression trends along tumor progression, we selected the top 1,000 genes with highest PCA loadings, and divided the means of normalized expression at week 8, 10 and 12 by that of week 6 to get relative expression ratios. We then used *k*-means clustering to group genes based on the ratio. We plotted the number of clusters against the total within cluster SS (sums of squares) and selected the cluster value at the first inflection point, which is 5.

### Quantitative real-time PCR (qPCR)

The qPCR was performed using the 2^−ΔΔCt^ method. The RNA was converted to cDNA using SuperScript III First-Strand Synthesis Kit (Invitrogen), followed by reactions using ABI StepOnePlus Real-Time PCR System instrument (Applied Biosystems). Each 10μl reaction included 5μl Taqman Fast Master Mix, 2.5μl RNAse-free water, 2μl cDNA (10 nM) and 0.5μl Taqman primer set. *Actb* was used as the internal control gene to give genes tested a relative fold change using the 2^−ΔΔCt^ method. The qPCR primers are by Taqman Gene Expression Assays, catalog number 4448892.

## Results

### Differentially expressed genes are enriched in cancer-related pathways and E2F targets

**Fig. 1:**
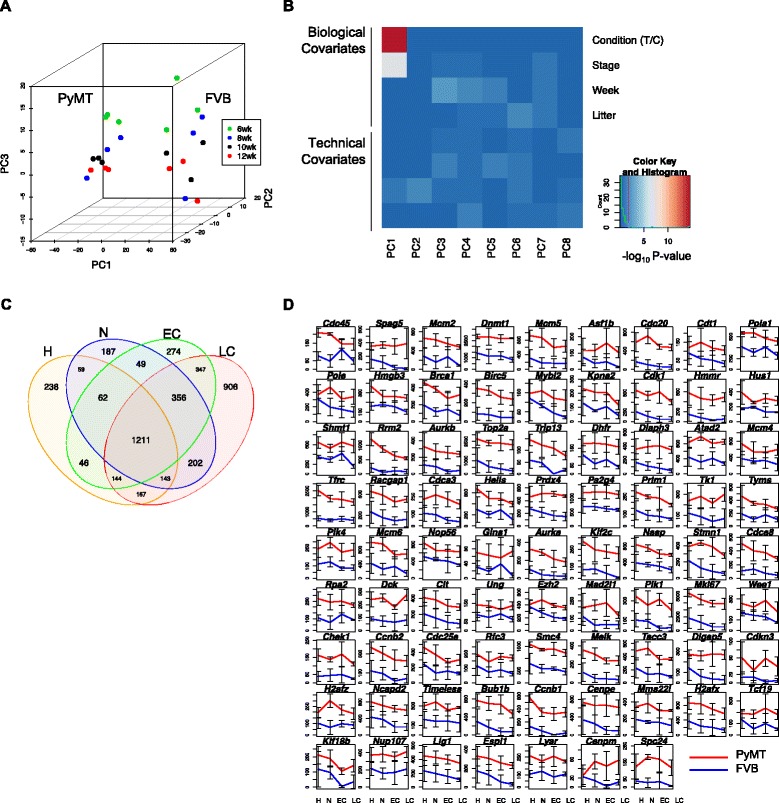
Differentially expressed genes along tumor progression. **a** PCA of PyMT and FVB samples. It shows the separation between tumor and control samples. **b** Biological and technical confounders’ contribution to PCs. The heatmap shows –log10 *p*-value of linear regression of top PCs on some known covariates. Red indicates significant correlation. The disease status, whether it is tumor or control, contributes most significantly to PC1 and PC2. No technical covariates exhibited correlation to any PCs. Plotted with the ggplot2 R package [80]. **c** Venn diagram of DEGs. A majority of the DEGs at each one of the four time points were also detected in at least one another time point. **d** Mean expression of 79 E2F-targeted DEGs at four stages. The red line denotes PyMT tumors, and the blue line controls. The normalized gene counts were plotted with the error bar showing the standard deviation. 79 targets were differentially expressed in late carcinoma stage (week 12). H, hyperplasia; N, adenoma/MIN; EC, early carcinoma; and LC, late carcinoma

To determine the transcriptional distinction among different cancer developmental stages, we first performed PCA analysis (Methods). The PyMT tumor samples are clearly separated from the FVB controls on PC1. There is also a temporal separation among the PyMT samples from various stages except for one adenoma/MIN sample (Fig. 1a, Additional file 1: Figure S1B). In Fig. 1b, we showed contributions to PCs from different biological and technical conditions as covariates; it is clear that only biological contribute to PCs, but not the technical ones. Disease condition and stage strongly contribute to PC1, mouse age contributes to PC3, and litter slightly contributes to PC2. This means that the variance separating PyMT tumors from FVB controls was the underlying biological factors, not the technical ones such as batch effect. In addition, we examined genes with top loadings on PC1 and found that only *Fhl1* and *Txnip* are cell cycle-related genes. This means that the difference we observed was mainly due to something other than cell cycle.

We then examined differentially expressed genes (DEGs) at each stage. At each time point (week 6, 8, 10 and 12), we compared three PyMT samples with three FBV controls (Methods), and identified 2,070, 2,269, 2,489, and 3,476 DEGs respectively (fold change > 2, FDR < 0.05, Table 1 and Additional file 2: Table S1). The last stage (late carcinoma) had the most DEGs and was with a big increase on the DEG number comparing to all other stages. We also found that more genes were down-regulated than up-regulated ((1,430, 1,486, 1,609, 2,205) vs. (640, 783, 880, 1,271) DEGs at each stage (Table 1). Among the union of all 4391 DEGs, a significant proportion of DEGs (27.2%, 1,211 genes) appeared at all four stages (Fig. 1c). This is consistent with a previous human study, which showed that gene expression in ADH, DCIS, and IDC are highly similar during tumor progression [4]. Yet, metastatic risk could be predicted based on gene expression profile of primary carcinoma [29, 30]. Among these 4,391 key genes, 92 genes were among the 289 breast cancer-related genes identified by MalaCards [5].

**Table 1:** Differentially expressed genes at four stages

Stage	Down	Up	Total
Hyperplasia	1430	640	2070
Adenoma/MIN	1486	783	2269
Early carcinoma	1609	880	2489
Late carcinoma	2205	1271	3476
Union	2642	1749	4391

Note: Numbers of DEGs identified at the four stages

**Fig. 2:**
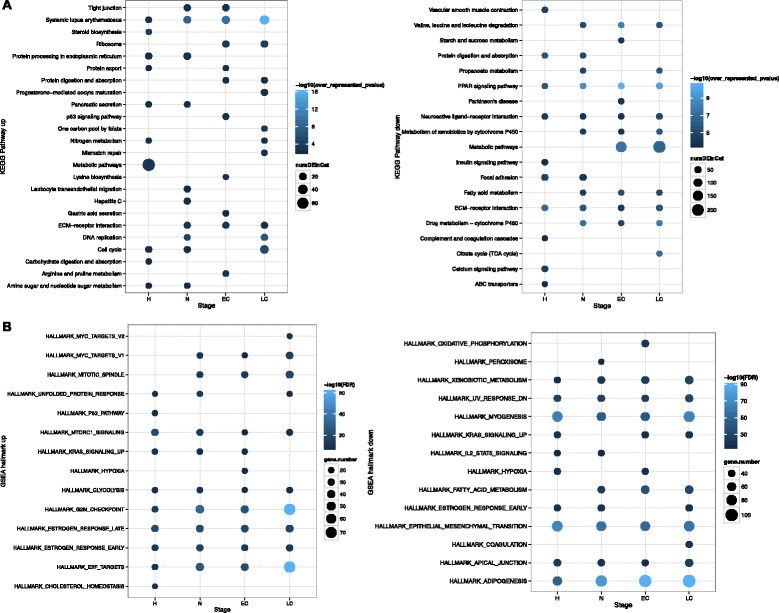
Pathway enrichment analysis. **a** KEGG enrichment of DEGs at four stages. **b** GSEA-hallmark enrichment. The top 10 terms with the most significant FDR at each stage were plotted. H: hyperplasia, N: adenoma/MIN, EC: early carcinoma, LC: late carcinoma. Color represents –log10 of FDR or over represented *p*-value, dot size represents the number of DEGs in each KEGG or hallmark term

We then analyzed REACTOME, KEGG, and GSEA-hallmark enrichment (Methods) of the DEGs identified at each stage. Of the KEGG pathways, the extracellular matrix (ECM) receptor interaction (KEGG:04512) and metabolic pathways (KEGG:01100) were enriched in the down-regulated DEGs; DNA-replication (KEGG: 03030) and cell cycle (KEGG: 04110) in the up-regulated DEGs (Fig. 2a). Of the REACTOME pathways, metabolism and extracellular matrix related terms were also enriched in the down-regulated DEGs; the up-regulated DEGs were enriched on cell cycle and DNA methylation (Additional file 3: Figure S2). Of the GSEA hallmarks, we uncovered enrichment on the E2F targets, G2M checkpoint, MTORC1 signaling in the up-regulated genes (Fig. 2b). We are mostly interested in the E2F targets because the transcription factors E2Fs mediate G1/S transition in cell cycles [31] and regulate tumor development and metastasis as indicated by an early study using the PyMT mice [32]. Interestingly, E2F2 was differentially expressed only in hyperplasia stage with an increase of 3.7 fold (FDR < 0.05) but not in other stages; the E2F targets enrichment was more significant in the later three stages, indicating a possible time lag for the transcription factors to function. We found 79 E2F targets as DEGs from week 12; their differential expression was much higher in the PyMT mice than in controls and this difference maintained throughout tumor progression (Fig. 1d). Many E2F targets showed a gradual reduction from week 6 to week 12 in both groups, suggesting a higher activation of development in early life, while some others maintained the high expression in PyMT mice, such as *Dnmt1*, *Diaph3*, and *Prdx4* (Fig. 1d). Their functional roles in processes other than cell cycles could also be important for tumor progression.

We found genes in P53 signaling pathway with increased expression at early carcinoma stage (Fig. 2a), for example *Rrm2*, which functions in DNA repair and damage prevention. *Rrm2* encodes a ribonucleotide reductase and was reported as an indicator of tamoxifen resistant in luminal patients as well as decreased survival in patients of all breast cancer subtypes [33]. In our study, the expression of *Rrm2* increased throughout the breast cancer development in PyMT mice (log2 fold change in hyperplasia, adenoma/MIN, early carcinoma, late carcinoma is 2.5, 4.0, 2.7, and 2.8, respectively) and may serve as potential biomarker for diagnosis as well as drug target.

In addition to terms related to cell cycle and proliferation, we also observed other significant enrichment such as PPAR (peroxisome proliferator-activated receptor) signaling (KEGG:03320) and drug metabolism- cytochrome P450 (KEGG:00982) (Fig. 2b). In late carcinoma, *Pparg* was among the 26 down-regulated PPAR pathway genes (*Cd36, Sorbs1, Pparg, Angptl4, Lpl, Fabp4, Acox1, Acadm, Me1, Cpt2, Slc27a1, Scp2, Adipoq, Acsl1, Fabp3, Ppara, Plin1, Cyp27a1, Acox3, Pltp, Pck1, Ilk, Aqp7, Rxrg, Cpt1c,* and *Nr1h3*); *Cyp2e1* was among the 17 down-regulated drug metabolism-related cytochrome P450 genes (*Mgst3, Ugt1a9, Adh1c, Fmo1, Gstz1, Aox1, Cyp2e1, Gsta3, Gstt2, Maob, Cyp2d6, Fmo2, Gstm1, Ugt1a5, Ugt1a3, Gstm2*, and *Aldh3b2*).

### Hub genes from co-expression network modules predict metastasis in human breast cancer datasets

**Fig. 3:**
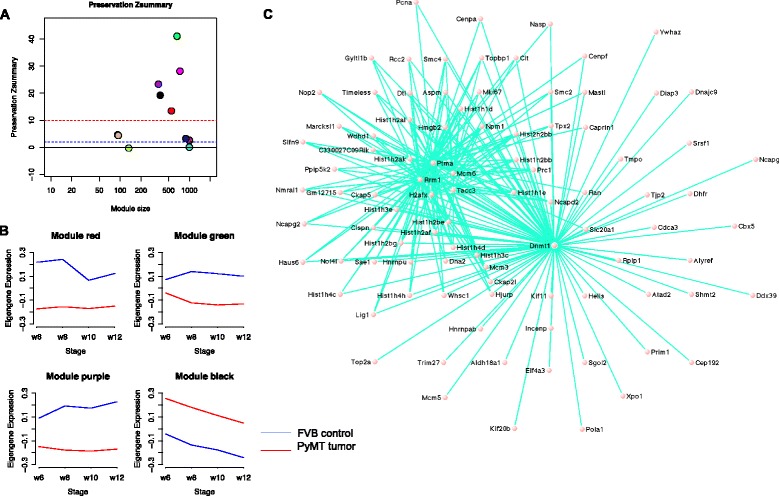
Weighted gene co-expression network analysis (WGCNA). **a** Module conservation. Modules above red line (Zsummary >10) were highly preserved, above blue line (Zsummary >2) were moderately preserved. **b** Co-expression connections in the black module. The connections are simplified to highlight 6 hub genes (*Dnmt1, Tacc3, Ptma, Mcm6, H2afx* and *Rrm1*) with annotation in KEGG pathways. For visualization purpose, only strong connections with topological overlap above a threshold of 0.2 are shown. **c** Module egigengene trajectories (the first principal component, PC1). Red lines are tumor, blue lines are control. Generally, genes in black module increased expression in tumors, and genes in other modules decreased expression

To identify genes expressed together as modules on a higher systems level, we explored coordinated transcriptional activities among genes in co-expression networks to reveal higher-order expression patterns and signatures of tumors. This network-based method is useful to detect potentially new biomarkers, and complements the identification of individual DEGs. We carried out a weighted gene co-expression network analysis (WGCNA). We first calculated Pearson’s correlation coefficients to measure gene-gene co-expression in our mouse samples. Then we used a topological overlap measure algorithm and identified 12 gene modules (Methods). Five of them are highly preserved (Fig. 3a), i.e., gene-gene co-expression in these five modules is similar in both PyMT tumors and controls. Among them, four modules showed significant difference between tumors and controls (Methods, Fig. 3b). Pathway enrichment showed that the modules are enriched with pathways of DNA replication (KEGG: 03030) and cell cycle (KEGG: 04110); focal adhesion (KEGG: 04510) and ECM receptor interaction (KEGG:04512); oxidative phosphorylation (KEGG: 00190) and TCA cycle (KEGG: 00020), and several metabolic pathways including insulin pathway (KEGG: 04910), respectively (Table 2). Genes in the black module (Additional file 1: Table S1) are involved in DNA replication and cell cycle hence may shed light on the molecular mechanisms of breast cancer in this mouse model. This module is also enriched with targets of E2F and targets of MYC (GSEA hallmark). Genes with the most connections (‘hubs’) in a module capture most of the expression variability and can be considered as the module signature. The top 20 hub genes in the black module are *Uhrf1*, *Cct3*, *Dnmt1, Kif11, Top2a, Ptma, Mcm6, Tpx2, Smc2, Rrm1, Mki67, Tacc3, Ncapd2, Prc1, H2afx, Hist2h2bb, Hist1h1d, Npm1, Hist1h3c,* and *Hist1h2bb* (Fig. 3c). Nine genes in this module – *Akt1, Brca2, Ccnd1, Dnmt1, Mki67, Palb2, Rrm1, Timeless,* and *Top2a* – are known human breast cancer genes according to the MalaCards database [5]; four of them are hub genes. Not surprisingly, we found an overlap between hub genes and previously identified DEGs, including *Dnmt1, Top2a, Tacc3, Mcm6*, and *Mki67.*

**Table 2:** WGCNA modules with high preservation

Module	Genes	GO enrichment (FDR < 0.05)	KEGG pathways enrichment (FDR < 0.05)
Green	663	Mitochondria, membrane	Oxidative phosphorylation, TCA cycle
Red	550	Extracellular matrix, cell adhesion	Focal adhesion, ECM-receptor interaction
Purple	357	Mitochondria, peroxisome	Metabolism pathways, insulin pathway
Black	372	Cell cycle, M phase, mitotic	DNA replication, cell cycle

Note: Gene numbers in each module with GO and KEGG term enrichment

The PyMT mice provide a very aggressive breast cancer model with a metastatic rate of over 90%. We hypothesized that genes whose expressions correlate with tumor progression may mark more aggressive tumors and thus can be used to predict metastasis risk. To test this hypothesis and to translate our model-based findings to the human disease, we assessed the impact of hub genes on distant metastasis-free survival (DMFS) using the Kaplan-Meier analysis by querying the Gene expression-based Outcome for Breast cancer Online (GOBO) [34]. Based on their high or low expression levels, ten hub genes – *H2AFX, KIF11, MCM6, MKI67, NCAPD2, PRC1, SMC2, TACC3, TOP2A, TPX2 *– separated breast cancer patients into high- and low-risk groups, which showed statistically significant difference in DMFS (Additional file 4: Figure S3). Using the ten genes together as a panel also gave a significant prediction but showed no increase on predicting power. In the purple module, among top 15 genes that we selected as module hubs, 12 (*PEX19, DHDH, ACSL1, ALDH6A1, PNPLA2, PPP2R5A, IVD, MME, ADIPOR2, ALAS1, CDO1*, and *CHPT1*) are non-cell cycle genes and as a gene panel can separate patients with different risks by Kaplan-Meier analysis (*p*-value < 0.05), while the other three cell cycle-related genes (*AKT2, INSR*, and *YWHAG*) cannot. Hubs from the other two models showed no significant ability for separation.

Note that many hub genes as E2F targets are involved in tumorigenesis. As an E2F target, *Dnmt1* (DNA methytrasferase 1) showed differential expression throughout tumor progression (expression fold changes in PyMT tumors vs. FVB controls during hyperplasia, adenoma/MIN, early carcinoma, and late carcinoma are 2.0, 2,5, 2.3, and 3.2 (FDR < 0.05), respectively). DNMT1 is crucial for the maintenance of DNA methylation, which causes gene silencing and is known to regulate many cancer-related genes including many tumor suppressors [35, 36]. These gene expression changes suggest a global methylation deregulation and a potential key role of DNA methylation in breast cancer. In addition, we have carried out an enhanced reduced representation bisulphite sequencing (ERRBS) of the same PyMT mouse model to examine DNA methylathion changes during its cancer progression process in another study to be published. *Mki67 (Ki67)* is a widely recognized marker for cell proliferation and is associated with breast cancer prognosis statistically [37]. *Top2a* codes for topoisomerase II Alpha, a key enzyme in DNA replication that regulates gene expression and cell division and whose amplification is a predictor for anthracycline treatment in breast cancer [38]. Another hub gene *Tacc3* plays roles in cell cycle, immune system development, and microtubule cytoskeleton organization. An up-regulation of *Tacc3* was observed in breast cancer and it was suggested that *Tacc3* might be a deregulator of DNA damage response and a predictor of survival for breast cancer patients [39]. *Mcm6* and *Ncapd2* are E2F targets as well, and little is known about their roles in breast tumor progression. *Mcm6* is one of the mini-chromosome maintenance proteins and has been suggested as a prognostic marker in melanoma [40]. In the black module, we found *Mcm2-6* and *Mcm9*. The *Mcm2-7* and *Mcm8-9* form protein complexes respectively to function in DNA replication initiation and DNA recombination repair [41, 42]. An early study showed a strong association between the over expression of *Mcm2-7* and the short survival in a breast cancer cohort [43] and suggested their roles in breast tumor progression. Here we added an additional layer of evidence. *Ncapd2* (non-SMC condensing I complex subunit D2) is involved in mitotic sister chromosome segregation [44] without notable involvement in breast cancer. We think its alteration may represent elevated cell mitosis in tumors like that of *Mki67*.

The green module showed a slight negative correlation between eigengene and tumor progression compared to controls. 20 genes from the TCA cycle pathway (*Acly, Aco2, Csl, Cs, Dlat, Dlst, Dld, Fh1, Idh2, Idh3a, Idh3b, Idh3g, Mdh1, Ogdh, Pdhb, Pdha1, Sdha, Sdhd, Suclg1,* and *Sucla2*) were found in the green module. This enrichment indicates a significant involvement of energy metabolism in tumor progression. In the purple module, we found *Eif4ebp1* with reduced expression in tumor. This gene is a eukaryotic translation initiation factor biding protein that functions in the insulin-signaling pathway. It inhibits the oncogene *Eif4e*, whose overexpression leads to human hepatocellular carcinoma development together with Ras activation [45].

### Time-course analysis identifies expression patterns of tumor progression and malignant transition

**Fig. 4:**
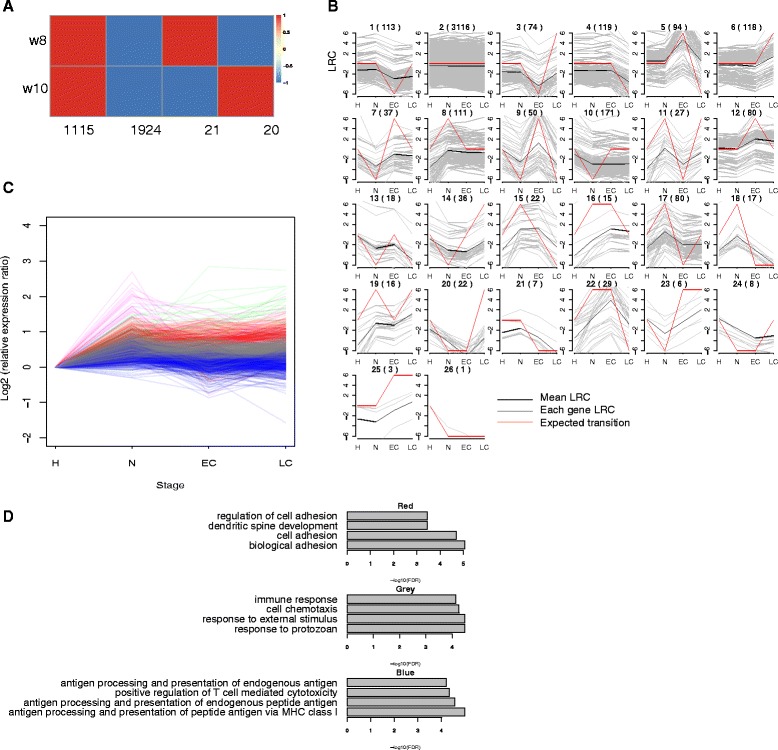
Time-course analysis. **a** Gene groups based on expression transition between week 8 (adenoma/MIN) and week 10 (early carcinoma); red with value 1 = increased expression, blue with value -1 = decreased expression. **b** A total of 26 groups of various transition statuses. The group 1 and group 12 were the two representative groups. The LRC (log2 ratio change) is plotted against the three transitions, red lines represent the assumed transitions with value of -1, 1 or 0; grey lines are individual genes; black lines are the median. Constant transition as 0, 1 and -1 indicate positive and negative changes respectively. Numbers are genes in each group. Genes in these two groups showed significant change from adenoma/MIN to early carcinoma, when the malignant transition happens. H: hyperplasia, N: adenoma/MIN, EC: early carcinoma, LC: late carcinoma. **c** Monotonic time course analysis identified three major groups of genes. **d** GO Biological Process terms enrichment of the three groups, plotted top four results

In PyMT mice, the malignant transition occurs between adenoma/MIN (week 8) and early carcinoma (week 10), so we first examined genes that showed fluctuation during this time. We classified the union of all DEGs in week 8 and week 10 (3,080 genes) into four groups based on their up- or down-regulation patterns. 21 genes exhibited up-/down-regulation and 20 genes exhibited the opposite down-/up-regulation (Fig. 4a). Genes with simple expression transition are surprisingly few. As an alternative, we defined a type of statistically transition (Methods). Based on their expression profiles, we clustered the union of 4,391 DEGs into 26 groups of transition patterns (because there was no gene showed all positive transition, left only 26 groups instead of 27 groups (Fig. 4b). As defined, most genes (3,116 genes in group 2) showed no change for all three transitions. Since the malignant transition happens between adenoma/MIN and early carcinoma, we were mostly interested in genes with a peak of either positive or negative change at the second transition. The 193 genes – 113 in group 1 and 80 in group 12 (Fig. 4b) – displayed this transition pattern. Enrichment (FDR < 0.05) revealed KRAS signaling up and estrogen response early (GSEA hallmark); Calcium signaling (KEGG: 04020) in these genes. The GSEA hallmark E2F targets including *Cdk1, Ccnb2, Plk1, Aurkb,* and *Spag5* were also enriched (FDR < 0.05) in the group 12. Moreover, we found *Erbb4* (log2 fold-change: 0.03, -0.18, 2.39, 2.05) in the group 12. *Erbb4* appears to be an oncogene in breast cancers [46, 47], and *Erbb2* has been suggested to help *Erbb4* carry out the oncogenic activities [46]. Interestingly, Ingenuity Pathways Analysis (Ingenuity® Systems, http://www.ingenuity.com) identified *Erbb2* as a potential upstream regulator (Additional file 5: Figure S4) in week 10 (early carcinoma). Taken together, these two genes may function simultaneously in malignant transition.

As a compliment to the transition analysis, we explored the continuous monotonic pattern in tumor samples to study genes with possible tumor maintenance functions (Methods). We first isolated 1,000 genes with highest PCA loading and divided the mean of normalized expression at each time point by that of week 6 to obtain the relative expression ratio. Using *k*-means clustering, we then separated these genes into five groups with three major ones (Fig. 4c). Genes in red group mainly function in cell adhesion (GO: 0007155) and biological adhesion (GO: 0022610), maintained high expression as tumor progress. Genes in the blue group play roles in the immune system such as antigen processing and presentation, showed decreased expression especially at early carcinoma stage. Genes in the grey group, enriched in response to external stimulus (GO: 0009605), showed no obvious change as tumor progressed (Fig. 4d).

### Novel genes involved in PyMT mouse model of breast cancer

Using R package RISmed, we mined PubMed for the functional relevance of the newly discovered genes in tumor development. We used RISmed to extract bibliographic data from PubMed, and examined if the official symbols of genes that we are interested in concurred with the key word “breast cancer” in article titles or abstracts. If so, such genes are considered to have functions related to breast cancer. Of the 4,391 DEGs, about half have no reported roles in breast cancer. Of the 79 E2F targets we identified in late carcinoma, 14 genes (*H2afz, Spc24, Cenpm, Gins1, Pola1, Prim1, Nop56, Kif18b, Mms22l, Lyar, Tcf19, Cdca3, Trip13, Ncapd2*) are not previously described in breast cancer. Among those five E2F targets correlated with malignant transition, *Cdk1* and *Plk1* were well studied in breast cancer, but much less so for *Ccnb2, Aurkb,* and *Spag5* (7, 10, and 3 reports, respectively)*.* Among the 79 E2F targets, *Hells, Hmgb3,* and *Cit* are of special interests because of their functions. HELLS interacts with DNMT3A and DNMT3B in the STRING protein-protein interaction database [48, 49], and is known to be involved in survival, *de novo* DNA methylation, and DNA methylation maintenance [50]. It also cooperates with HDAC1, HDAC2, and DNMTs to silence transcription [51]. This is consistent with our finding of increased *Dnmt1* expression and global gene down-regulation. HMGB3 promotes cell proliferation in bladder cancer [52] and interacts with TOP2A and TOP2B, two targets for some anticancer agents. CIT, a serine/threonine-protein kinase, functions together with *Kif14* (its expression increased by more than four folds in the TCGA breast cancer samples) in cell division. In summary, we propose some less studied genes as potential new players in breast cancer progression.

**Fig. 5:**
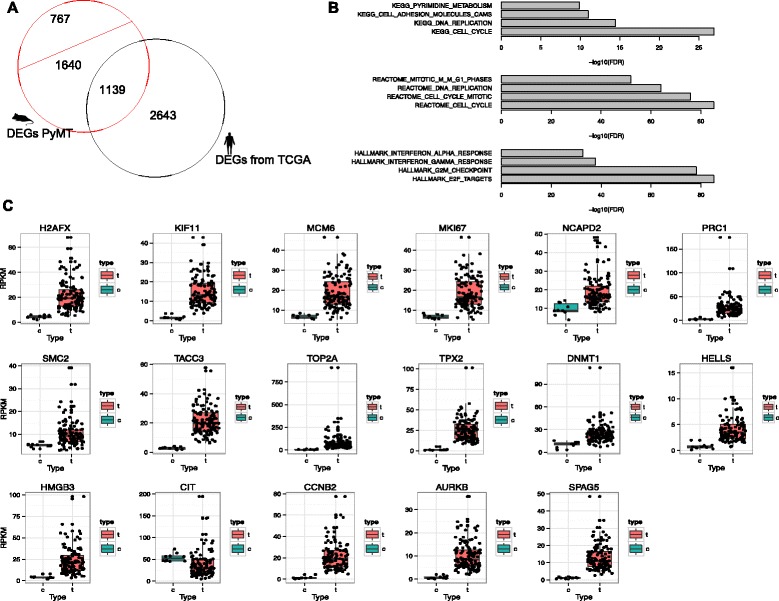
Analysis on the TCGA data. **a** Comparison with human TCGA data. Black circle is the 3,782 DEGs identified in TCGA human breast carcinoma samples; intersect region is the 1,139 DEGs identified in both PyMT and human late carcinoma samples. **b** KEGG, REACTOME and GSEA hallmark enrichment of DEGs in the TCGA data. **c** Expression of the 17 E2F targets of interests in the TCGA data; “t” = tumor, “c” = control

To investigate DEGs identified from PyMT mouse in the context of human, we first used publically available RNA-sequencing data of human breast invasive carcinoma (BRCA) from the TCGA Research Network (http://cancergenome.nih.gov/). We downloaded 115 luminal B subtype BRCA patients data (classified by the PAM50 model [53] in this TCGA paper [14]), which most closely resembles the PyMT mouse model [9]. Because we had three samples at each stage, we only used 10 patient samples with 10 matched adjacent normal mammary tissue as controls thus not to inflate DEGs detecting power. Their Level3 TCGA BRCA RNA-sequencing raw gene counts were processed the same way as for our PyMT data; 3,782 DEGs were identified (FDR < 0.05, fold change > 2). Among those 3,476 DEGs from PyMT late carcinoma stage, 2,779 genes were with human homologues, and 1,139 of them were significantly up- or down-regulated in both PyMT mouse and human, 918 genes in the same direction in both human and mouse (Fig. 5a). Human homologs gene list was downloaded from MGI [41, 54, 55] (Mouse Genome Informatics, http://www.informatics.jax.org/). Among these DEGs from TCGA samples, the up-regulated ones were enriched in cell cycle (REACTOME), E2F targets and G2M checkpoint (GSEA hallmark, Fig. 5b), similar to the enrichment profile of the PyMT mice. There were 100 E2F targets in the 3,782 DEGs out of a total of 200 E2F targets defined by GSEA, a very significant enrichment. We observed significant (*p*-value < 0.05, *t*-test) increased expression of the genes of interests discussed previously in the TCGA luminal B patients except for *Cit* (Fig. 5c). Breast cancers in human and PyMT mice are likely to share similar expression of certain candidate genes. The aforementioned expression analysis of the TCGA data validated our RNA-sequencing results.

To see how the differences in expression are reflected on the gene product level, we queried The Human Protein Atlas [56] for RNA expression and protein staining scores in the metastatic breast cancer cell line MCF7. Of the ten hub genes tested by the Kaplan-Meier analysis, seven showed significant protein expression (Additional file 2: Table S6).

We also compared our results with other studies of PyMT breast cancer. In a previous study, Andrechek et al [57] compiled various PyMT datasets from the GEO database and identified 4,184 DEGs between PyMT mouse tumor and normal mammary gland. Comparing this set of genes with our 3,476 DEGs from the late carcinoma stage, we found an overlap of 2,069 genes with expression changes in the same direction. To see how our DEGs behave in other mouse models, we compared the same 3,476 DEGs from our study with 4,018 DEGs identified in the same study [57] between carcinoma in MMTV-Neu mice (a mouse model of breast cancer with pulmonary metastasis) and normal mammary gland. We found an overlap of 1,642 genes with expression change in the same direction.

To verify our RNA-sequencing results, we performed qPCR on the same samples used for sequencing. We confirmed increased expression of *Top2a* and *Dnmt1* in all four stages (*p*-value < 0.05, Additional file 6: Figure S5A). To validate that our findings are reproducible, we also carried our qPCR on independent samples from PyMT and FVB mice not used for sequencing. We selected six genes showing increased expression in the PyMT mouse from all stages, and observed similar expression in the qPCR results (Additional file 6: Figure S5B).

## Discussion

A major challenge in breast cancer study is to understand the molecular mechanisms of malignant progression. Mouse models have been widely used to characterize the molecular events in breast cancer. Compared to early microarray-based gene expression studies of PyMT mouse carcinoma [58–60], we demonstrated for the first time transcriptional changes associated with distinct stages in breast cancer progression using RNA-sequencing and observed temporal global mRNA expression deregulation in the PyMT mouse model. Some previous studies used PyMT mouse model to identify signature or biomarkers of tumor virulence [61], or residual tumors and lung metastases [58]. Another study [57] has indicated that highly expressed genes in PyMT mouse are enriched for gene that predict metastasis using the van’t Veer gene set [30]. Many more studies compared various breast cancer mouse models as well as human samples to get mouse model classes and general molecular features of the disease [9, 57, 59]. But none focused on the changes during tumor progression.

We observed that most DEGs identified in the late carcinoma stage first appeared in the much earlier hyperplasia stage. This is consistent with previous human cancer studies, which reported substantial similarity of transcriptional profiles in various pathological stages [4] and suggested that expression profiles of early primary tumors may tell progressive potential [29, 30]. Similar to our findings, earlier investigations with microarrays also observed that more genes were down-regulated in both PyMT mouse [9] and human luminal B subtype [14]. Many of the significantly enriched pathways are actually part of the big “pathway in cancer” (KEGG:05200), such as ECM-receptor interaction and focal adhesion. Although not significant enriched in our data, the PyMT oncogenesis causal pathway PI3K-AKT signaling is also included in the “pathway of cancer” so is linked to many of those enriched pathways. Metabolism deregulation are widely recognized in cancer, such that high calorie diet elevates cancer risk, especially on glucose metabolism [62]. In both DEGs and WGCNA module genes, we found enrichment of many metabolic related terms and pathways, including the TCA cycle pathway.

With the co-expression network analysis, we identified genes that function together. Because co-expression networks derived from RNA-sequencing data exhibit higher correlation and hence higher connectivity than ones derived from microarrays [63], gene modules found in this study could better explain molecular mechanisms of the disease progression. One module with increased gene expression in tumor is enriched with DNA replication and cell cycle genes. Module hub genes are not only potential prognosis biomarkers of the human disease demonstrated by our Kaplan-Meier analysis with data from human patients but also potential risk factors underlying the molecular mechanisms of human breast cancer. Some hubs in this module are known cancer-related genes, however some have not been identified as oncogenes or cancer biomarkers so far, such as *Mcm6* and *Ncapd2*. Although many hub genes can predict future metastasis with statistical significance, the high and low risk groups show only small difference on risk for metastasis. It is possible that individual genes have only limited predictive power and more genes need to be combined into gene panels for finer separation of different risk groups as shown in many gene signature studies [29, 30]. The module enriched in oxidative phosphorylation and the TCA cycle showed decreased expression in tumor, which is consistent with the facts that cancers switch from oxidative phosphorylation to glycolysis due to hypoxia and rely less on the TCA cycle [64]. The reduced expression of TCA cycle genes in carcinoma stage has been reported before in the TAG mouse model [57]. It is very interesting that we observed metabolic changes in the early stages; they can be potential biomarkers for diagnosis. Nonetheless, we may not entirely rule out the possibility that the changes were due to hypoxia induced by the aggressive development for this particular mouse model. Focal adhesion, ECM-receptor interaction, and insulin pathway in other modules were also discovered in previous cancer DEGs studies.

E2F factors, including E2F1 to E2F3A, are well-known transcription activators in cell cycle [65–67], apoptosis, and proliferation [68]. Although we did not detect significant variation in their expression, possibly due to low expression levels, their target genes were enriched in DEGs as well as in co-expression modules. Recent studies have unveiled other functions of E2Fs beyond cell cycle [69]. E2F1 expression is suggested to correlate with esophageal squamous carcinoma progression [70]. Its knockdown reduced invasion potential but not proliferation [71]. E2Fs have been associated with relapse-free survival time in Myc-induced tumors [72]. They mediate tumor development and metastasis by regulating the expression of genes involved in angiogenesis [73], such as *Vegfa, Cyr61,* and *Angpt2*, and thus remodeling cell survival and ECM [32]. Besides, among 200 E2F target genes, there are 34 cell cycle genes and 22 DNA replication genes. Because the overlap between E2F targets and proliferation genes is only marginal, our enrichment result on E2F targets reflects more than a mere effect on proliferation. Although the involvement of E2Fs and their targets are well reported, here we proposed some new targets whose functions in breast cancer have not been documented. More importantly, we studied the expression of those E2F target in the early stages of tumor progression to investigate their potentials for cancer early diagnosis. The important roles that E2Fs play in metastasis warrant further investigation of their novel target genes that were discovered in this study. Moreover, the differential expression of *Hells* and *Dnmt1* suggests a crucial role of DNA methylation in tumor development.

The enrichment analysis of DEGs confirmed the importance of cell cycle process in the PyMT breast cancer tumorigenesis. Indeed, cell cycle and proliferation genes are strong predictors of metastasis [1]. Nevertheless, we also found DEGs enriched in other pathways such as PPAR signaling throughout tumor progression. PPAR signaling commonly regulates fatty acids metabolism and energy homeostasis and is part of the extensive “pathways in cancer” as defined by KEGG. There is emerging evidence of new cellular functions such as cell differentiation and tumorigenesis for PPAR signaling [74–76]. PPARγ, encoded by *PPARG*, is involved in multiple types of cancer including colonic tumor and breast tumor [74–76]. In breast cancer, PPARγ promotes terminal differentiation of malignant breast epithelial cells, and its activation triggered by drugs like antidiabetic thiazolidinedione (TZD) is associated with reduced cell growth and less malignancy [76]. Another down-regulated pathway in PyMT was drug metabolism-cytochrome P450. Cytochrome P450 (CYP) genes are responsible for phase I drug metabolism and are key enzymes contributing to tumorigenesis through metabolic activation of precaricinogenes [77]. As drug metabolizing enzymes, they also have crucial impact on anticancer drug treatment. CYP2E1, a feature gene in this pathway, was reported to inhibit cell migration in breast cancer cell lines on ectopic expression [78]. Taken together, some key genes from these non-cell proliferation pathways may also regulate tumorigenesis, and thus other genes in these pathways are worth further investigation.

Even though PyMT mouse mimic the human disease, expression levels of some oncogenes vary between the two species [79]. The PyMT oncoprotein is not expressed in human and the lung-specific metastasis nature in PyMT mice may lead to a not generalized molecular signature of breast cancer metastasis. Despite all the limitations, PyMT mouse model is widely appreciated in breast cancer research.

## Conclusions

We found remarkable expression similarity among hyperplasia, adenoma/MIN, early and late carcinoma samples, meaning genes altered in the late stages showed trace in the beginning of tumor progression. It is suggested that we may use early stage expression profile to help diagnosis and treatment. In addition, some E2F target genes identified by differential expression analysis, co-expression network analysis and time-course analysis may promote tumor progression and are new candidate for breast cancer that worth further investigation.

## Additional files

Additional file 1:
**Figure S1.** Quality controls. (A) Sample quality control. Histopathology images with H&E staining showed typical features of various stages (*40). (B) PCA plot without cell cycle and DNA replication genes. Compared with Fig. 1a, the similar result indicates that proliferation genes were not major contributors to PCs. (DOC 216 kb)
Additional file 2:
**Table S1–S4.** DEGs from the four stages. **Table S5.** Genes in the WGCNA modules. **Table S6.** RNA expression and protein staining scores from The Human Protein Atlas. (XLSX 181 kb)
Additional file 3:
**Figure S2.** REACTOME enrichment of DEGs at the four stages, the top 10 terms with the most significant FDR at each stage were plotted. H: hyperplasia, N: adenoma/MIN, EC: early carcinoma, LC: late carcinoma. (DOC 122 kb)
Additional file 4:
**Figure S3.** Kaplan-Meier analysis. The analysis was carried out with each individual hub gene and ten hub genes together as well. Patients were separated into two groups based on high (red) or low (grey) expression of each hub gene; Kaplan-Meier estimator showed significant difference in distant metastasis free survival (DMFS) between the two groups (*p*-value < 0.05). In the last plot, all ten hub genes were used together. (DOC 132 kb)
Additional file 5:
**Figure S4.** Upstream regulator of ERBB2 at week 10 (early carcinoma) as predicted by the Ingenuity Pathways Analysis (Ingenuity® Systems, http://www.ingenuity.com). (DOC 132 kb)
Additional file 6:
**Figure S5.** qPCR verification and validation of gene expression. (A) qPCR verification using the same RNAs sent for sequencing. (B) qPCR validation using RNAs from independent samples from PyMT and FVB mice, not used for RNA-sequencing. Asterisk “*”: *p*-value <0.05. C: controls; T: tumors. (DOC 75 kb)

## Abbreviations

CYP: Cytochrome P450
DEG: Differentially expressed gene
ECM: Extracellular matrix
EMT: Epithelia mesenchymal transition
LRC: log2 ratio change
mRNA: Messenger RNA
PyMT: Polyoma middle T
TZD: Thiazolidinedione
