# Supplementary material for: Transcriptomic dynamics of breast cancer progression in the MMTV-PyMT mouse model
Source: BMC Genomics. 2017 Feb 17;18:185. doi: 10.1186/s12864-017-3563-3 (PMC5316186; doi:10.1186/s12864-017-3563-3)

Click here to download Figure figs6.pdf

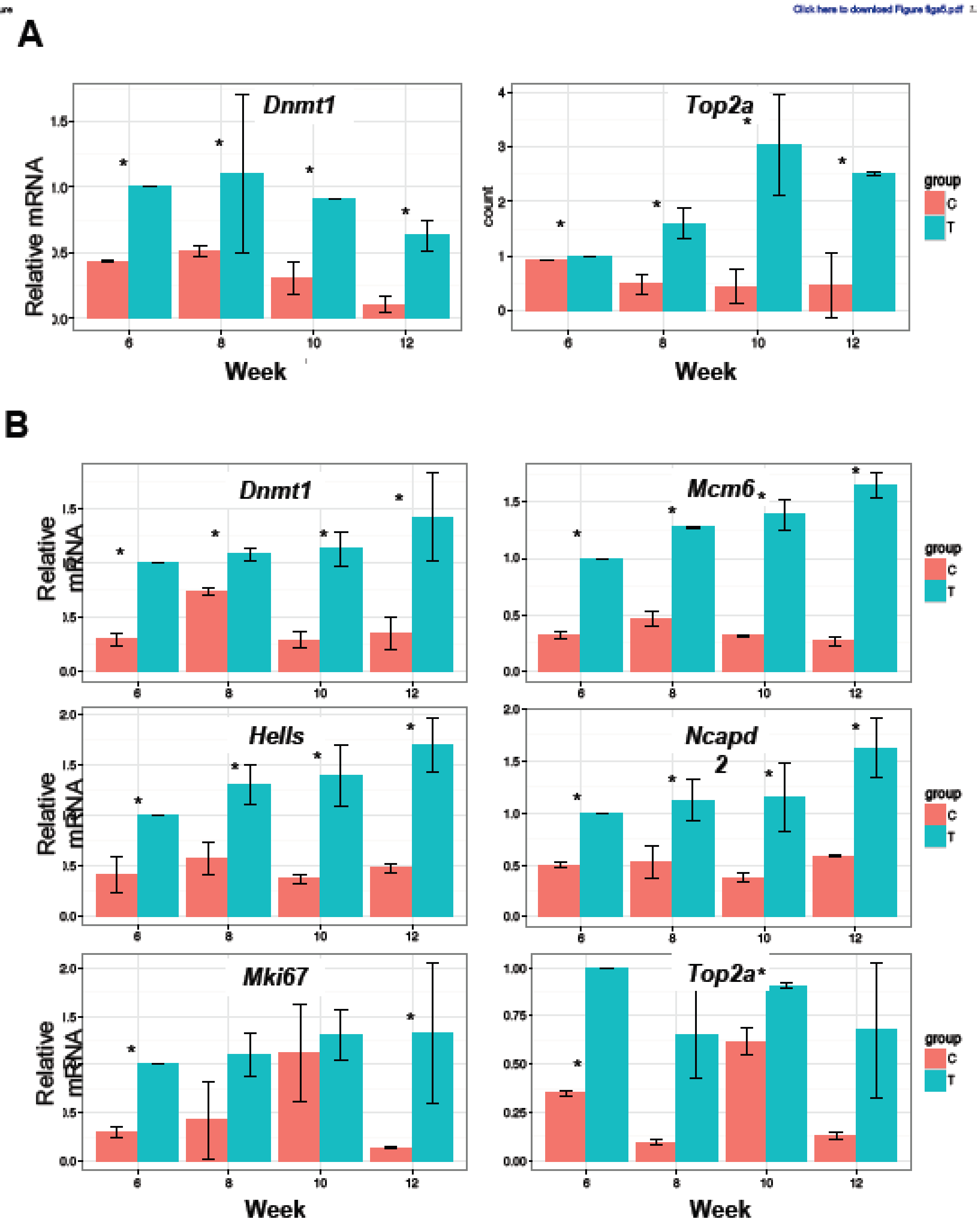

Supplement: Supplementary file 5 [file 12864_2017_3563_MOESM6_ESM.doc]
